# Early Response of Rhizosphere Microbial Community Network Characteristics to Thinning Intensity in *Pinus massoniana* Plantations

**DOI:** 10.3390/microorganisms13061357

**Published:** 2025-06-11

**Authors:** Size Liu, Haifeng Yin, Yu Su, Xianwei Li, Chuan Fan

**Affiliations:** 1 College of Forestry, Sichuan Agricultural University, Chengdu 611130, China; size_leo@126.com (S.L.); fanchuan01@163.com (C.F.); 2 Sichuan Academy of Forestry, Chengdu 610081, China; 3 Research Institute of Tropical Forestry, Chinese Academy of Forestry, Guangzhou 510520, China; yhfeng312@163.com; 4 Guangzhou Institute of Forestry and Landscape Architecture, Guangzhou 510405, China; yusu110@163.com; 5 Key Laboratory of State Forestry Administration for Forest Resources Conservation and Ecological Security in Upper Reaches of Yangtze River, Sichuan Agricultural University, Chengdu 611130, China

**Keywords:** forest thinning, rhizosphere microorganism, co-occurrence network, keystone taxa

## Abstract

Rhizosphere microorganisms mediate the material exchange and chemical cycling between plant roots and soil. However, the response mechanisms of the rhizosphere microbial community, especially its co-occurrence patterns, to thinning remain poorly understood. We investigated the rhizosphere microbial communities of *Pinus massoniana* under different thinning intensities, including control (CK, 0%), light-intensity thinning (LIT, 10%), moderate-intensity thinning (MIT, 30%), and high-intensity thinning (HIT, 50%). Basic taxonomic information was obtained through high-throughput sequencing, while R software was utilized to identify thinning-sensitive operational taxonomic units (tsOTUs), construct co-occurrence networks, and perform other statistical analyses. Although no discernible patterns were observed in α-diversity changes, the Kruskal–Wallis test indicated that season was the primary factor driving α-diversity variation. Meanwhile, thinning intensity significantly shaped the rhizosphere microbial community structures, with each intensity harboring a specific tsOTUs subset. Although the top three modules of the meta-co-occurrence networks in summer and winter exhibited consistent tsOTU composition, winter triggered changes in network connectivity. Regardless of summer or winter, the number of network nodes under MIT was the highest. Additionally, after thinning, the relative abundances of most keystone taxa declined; however, MIT facilitated the enrichment of certain keystone taxa. Collectively, thinning profoundly shapes microbial community composition and network characteristics. Moderate thinning intensity may represent the optimal thinning intensity for the studied *P. massoniana* plantations.

## 1. Introduction

The rhizosphere, typically defined as the region within 0.5–4 mm from the surface of plant roots, represents a sensitive zone for the intricate exchange of substances and chemical cycling between plant roots and soil [1,2,3]. The active microbial communities in the rhizosphere mediate ecological processes such as organic matter decomposition, nutrient cycling, and energy flow at the root–soil interface [4,5]. Therefore, they play a crucial role in forest ecosystems.

Thinning, as a common forest management practice, regulates forest ecosystem structure and enhances forest ecological functions. It has also been demonstrated to have the ability to impact the assembly mechanisms of rhizosphere microorganisms [6,7,8]. Thinning alters root production, biomass, and turnover rate, potentially driving a shift in the composition of rhizosphere sediments and inevitably affecting the rhizosphere environment [9,10]. At the same time, understory vegetation renewal after thinning leads to changes in soil physicochemical properties and substrate input, resulting in the alterations of microbial community in the bulk soil, indirectly affecting the recruitment of soil microorganisms by the rhizosphere of trees [11]. Numerous studies have demonstrated that rhizosphere microorganisms are more sensitive to management measures than bulk soil microorganisms [12,13,14]. Therefore, it is vital to consider the rhizosphere when evaluating the impact of thinning on the underground microbial community in forest ecosystems. However, due to limitations such as research subjects, study areas, and thinning gradients, there is still a lack of research regarding the effects of thinning on rhizosphere microbial communities.

Previous researchers have utilized statistical methods borrowed from macroecology to conduct extensive studies on forest soil microbial communities, mainly addressing fundamental ecological questions, such as understanding how different forest management practices affecting the α- and β-diversity of microbial communities [15,16,17,18]. However, interactions among taxonomic groups and their connection with ecological processes are frequently overlooked. A co-occurrence network constructed based on the correlated relationships among different microbial taxa can reflect the stability of microbial communities and reveal the functions and characteristics of the communities through its topological characteristic parameters (such as modularity, centrality, network nodes, and edges) [19,20,21]. This approach has been extensively applied in the research on soil microbial assemblage processes [22,23], providing a novel lens to explain several fundamental issues that remain unresolved in microbial ecology theory. The first is the identifications of key taxa. Key taxa are pivotal in sustaining the structural stability of microbial communities [13,24,25]. Overlooking their identification may erroneously suggest community fragmentation in research outcomes [26]. Another important issue is the significance of taxonomic richness for supporting the structure and function of microbial communities. Most of the literature studies currently focus on dominant species in soil ecosystems [27,28]. However, some low-abundance taxa are also significantly involved in ecosystem function and are reported to be keystones of the community [23,29,30,31].

*Pinus massoniana*, as an important fast-growing timber species in the subtropical and northern tropical regions of China, has been extensively planted for afforestation on barren mountains due to its tolerance to infertile soil conditions and heliophilous characteristics [7,32,33]. However, due to the lack of systematic and periodic silvicultural practices, the pure forests that have been established have exposed problems such as single differentiation of microenvironment and poor stress resistance, which seriously affect the development and reproductive environment of trees [34]. Previous studies have shown that thinning can alter soil quality, soil microbial communities, and fine root turnover in *P. massoniana* plantations [35,36,37,38]. However, there is a lack of research on the impact of thinning on the rhizosphere microbiota of *P. massoniana*, particularly regarding the study of microbial network characteristics. Therefore, this study focused on near-mature *P. massoniana* plantations with different thinning intensities. By constructing co-occurrence networks of rhizosphere microorganisms, our objectives were to (1) determine the initial responses of rhizosphere microbial diversity, composition, and network patterns to thinning in growth and dormancy periods; and (2) investigate the assembly mode of thinning-sensitive OTUs (tsOTUs) and the composition of keystone taxa.

## 2. Materials and Methods

### 2.1. Study Design

The study was conducted in *P. massoniana* plantations on Jinzi Mountain, Yuntai town, Pingchang County (31°37′06″–31°37′20″ N, 107°14′40″–107°15′03″ E), which is located in the northeast Sichuan Basin. This region belongs to the stepped valley landform, with an altitude of 710–730 m. The region is characterized by a subtropical humid monsoon climate with a mean annual temperature of 16.8 °C, precipitation of 1138.2 mm, 1365.5 daylight hours, and a frost-free period of 298 days each year. The soil in this study area is classified as yellow soil, with a depth ranging from 30 to 40 cm, and pH ranging from 4.29 to 5.13.

The *P. massoniana* plantations were established in 1991. The canopy density of plantations was as high as 0.8, average DBH was 17.5 cm, average height was 17.1 m, and stand density was 1500 trees/hm^2^. There was no current management of the plantation, and the understory vegetation mainly relied on natural regeneration, with low plant diversity. The understory dominant shrubs were *Myrsine africana*, *Eurya loquaiana*, and *Rhododendron simsii*, and the dominant herbs were *Miscanthus sinensis*, *Dicranopteris dichotoma*, and *Pteridium aquilinum*.

In June 2018, the stands with generally similar conditions of vegetation and landform were selected for thinning in the study area. The four treatments included no thinning (control; CK), 10% of the trees removed (low-intensity thinning; LIT), 30% of the trees removed (moderate-intensity thinning; MIT) and 50% of the trees removed (high-intensity thinning; HIT). Three replicate plots (30 × 20 m) were built for each treatment. In order to lessen latent edge effects, a buffer zone (10 m) was set around each plot. The plots were separated by 100 m from each other. Basic stand characteristics are shown in Table 1. The slope of each plot is less than 5°, so the influence of slope direction can be disregarded.

Given the potential disturbance to the soil environment caused by machinery, we opted for manual thinning and transportation of felled trees. Thinning was implemented using selective cutting method to ensure the uniform distribution of retained trees in the plots, and the felled trees were removed from the plots.

### 2.2. Sample Collection

Sampling was conducted five years after the completion of thinning in the plantations, at which point the canopy had achieved closure and the structure had stabilized. The rhizosphere dynamics of the trees had reflected improvements in growth conditions following the thinning measures [33].

In July 2023 (summer) and January 2024 (winter), we randomly selected five trees per plot as the sampling objects. From each selected tree, three primary lateral roots were carefully dug out and traced back to the tree to confirm their origin. Tertiary fine roots were removed from these lateral roots and shaken over a sieve to remove loose soil [39]. The collected fine roots were brought back to the laboratory immediately, stored at 4 °C.

In the laboratory, roots from the same plot were washed with 1 × PBS to remove the adhering rhizosphere soil. The wash solution was collected into 50 mL tubes and centrifuged for 10 min at 10,000× *g* to collect the precipitates. The resulting precipitates constituted twenty-four rhizosphere samples (four treatments × three replicates × two seasons) for subsequent microbial sequencing.

### 2.3. DNA Extraction and Sequencing

DNA was extracted using HiPure Soil DNA Kits (Magen, Guangzhou, China), and its quality was monitored by 1% agarose gel electrophoresis and by using a NanoDrop spectrophotometer (NanoDrop2000, Thermo Fisher Scientific, Wilmington, DE, USA). Qualified DNA was stored at −80 °C for subsequent analysis. The V3–V4 hypervariable region fragment of the bacterial 16S rRNA gene was amplified using primers 341F (5′-CCTACGGGNGGCWGCAG-3′) and 806R (5′-GGACTACHVGGGTATCTAAT-3′), whereas the internal transcribed spacer (ITS) fungal region was amplified using primers ITS3_KYO2 (5′-GATGAAGAACGYAGYRAA-3′) and ITS4 (5′-TCCTCCGCTTATTGATATGC-3′). The amplicons extracted from 2% agarose gels were purified using AMPure XP Beads (Beckman Coulter, Inc., Brea, CA, USA) and then quantified using an ABI StepOnePlus Real-Time PCR System (Life Technologies, Foster City, CA, USA). Purified amplicons were pooled in equimolar amounts and paired-end sequenced (PE250) on an Illumina HiseqTM 4000 by Gene De novo Biotechnology Co., Ltd. (Guangzhou, China).

### 2.4. Bioinformatic Analysis

The FASTP v.0.18.0 was used to remove low-quality sequences. Paired double-ended reads were spliced into a raw tag using FLASH v.1.2.11. The splicing condition was that the minimum matching length was 10 bp and the allowed mismatch rate of overlapping areas was 2%. The raw tags obtained by splicing refer to the quality control process of QIIME v.1.9.1 and were filtered to obtain effective tags. All effective tags were clustered at 97% similarity for identification of OTUs. The cluster was conducted with UPARSE v.9.2.64. The taxonomic assignment of OTUs was determined using the UNITE database (https://unite.ut.ee/) (accessed on 15 September 2024), and the RDP (Ribosomal Database Project) database (http://rdp.cme.msu.edu/) (accessed on 16 September 2024).

### 2.5. Statistical Analysis

All statistical analyses were performed using R software (version 4.3.2). The α-diversity indices were calculated using the “vegan” package (version 2.6-6) based on OTUs abundances. The Kruskal–Wallis test was performed to assess the differences in alpha diversity indices among microbial communities across different seasons and thinning intensities. The filtered OUT sequence counts were normalized using the “Trimmed Mean of M-values” (TMM) method in the “edgeR” package (version 4.3.1) and then expressed as counts per million (CPM) for relative abundance. Only OTU with at least 12 sequences in 24 samples were retained for further analysis. Unconstrained Principal Coordinates Analysis (PCoA) and Constrained Analysis of Principal Coordinates (CAP), based on Bray–Curtis dissimilarity, were performed using the “phyloseq” package (version 1.48.0) to compare differences among microbial communities. The effects of thinning and season on microbial community structure were tested using Permutational Multivariate Analysis of Variance (PERMANOVA) in the “vegan” package (version 2.6-6).

For all retained OTUs, we performed indicator species analysis using the R package indicspecies and likelihood ratio tests (LRT) using the R package edgeR. By synthesizing the results of these two analyses, we identified OTUs that exhibited significant responses to thinning and defined them as thinning-sensitive OTUs (tsOTUs). The bipartite network of tsOTUs was visualized using the “igraph” package (version 2.0.3).

The co-occurrence network was built using the method described by Hartman et al. [13]. Briefly, OTUs were selected for network construction based on pairwise comparisons, where each pair of OTUs had to exhibit Spearman correlation coefficients > |0.7| and adjusted *p* values < 0.05. We constructed individual bacterial and fungal networks, as well as meta-occurrence networks by combining bacteria and fungi, and subsequently calculated the topological network characteristics. For meta-occurrence networks, we further calculated the number of connections between bacteria and fungi, and employed the greedy optimization of modularity algorithms in the R package igraph to identify network modules with high edge density. The keystone OTUs in each network were defined as the top 1% of node degree values.

## 3. Results

### 3.1. Diversity of Rhizosphere Microbial Community

After filtering and removal, we obtained 1,710,152 bacterial sequences and 2,514,414 fungal sequences, which were divided into 3820 bacterial OTUs and 2756 fungal OTUs, respectively. The numbers of bacterial OTUs in summer and winter were 3190 and 2623, respectively, which were higher than fungal OTUs of 2041 and 1728.

The Chao index reflects species richness, whereas the Pielou index reflects species evenness. The two diversity indices exhibited different patterns of variation between different seasons and different thinning intensities (Figure S1). The Kruskal–Wallis test revealed extremely significant differences (*p* < 0.001) in the Chao and Pielou indices of bacterial communities and the Chao indices of fungal communities between two seasons. In contrast, no significant differences (*p* > 0.05) were observed in α-diversity indices among various thinning intensities (Table 2). Based on the above findings, it can be inferred that in the short term after thinning, thinning intensity did not significantly alter the rhizosphere microbial α-diversity, and season remained the dominant factor driving changes in α-diversity.

**Figure 1:**
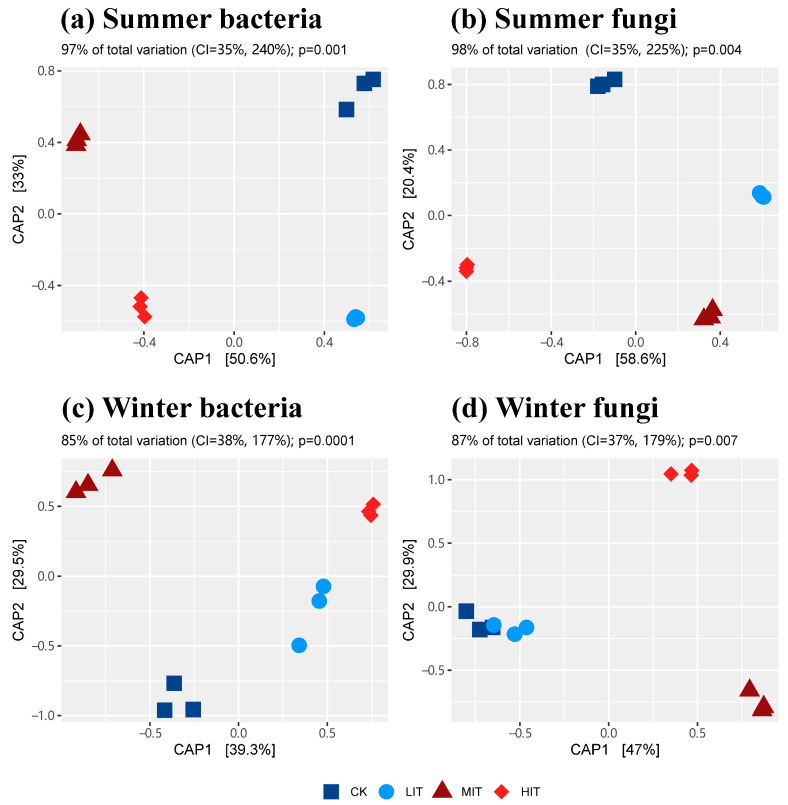
Constrained analysis of principal coordinates (CAP) of bacteria and fungi in summer and winter based on Bray–Curtis distance. CK, control; LIT, low-intensity thinning; MIT, moderate-intensity thinning; HIT, high-intensity thinning.

Regardless of summer or winter, rhizosphere bacterial and fungal communities formed distinct clusters under different thinning intensities (Figure 1 and Figure S2). Thinning explained 97% and 98% of the summer bacterial and fungal CAP-based Sorting, respectively, and 85% and 87% of the winter bacterial and fungal CAP-based Sorting. PERMANOVA also confirmed the significant effects of thinning and season on rhizosphere microbial communities (Table S1).

**Figure 2:**
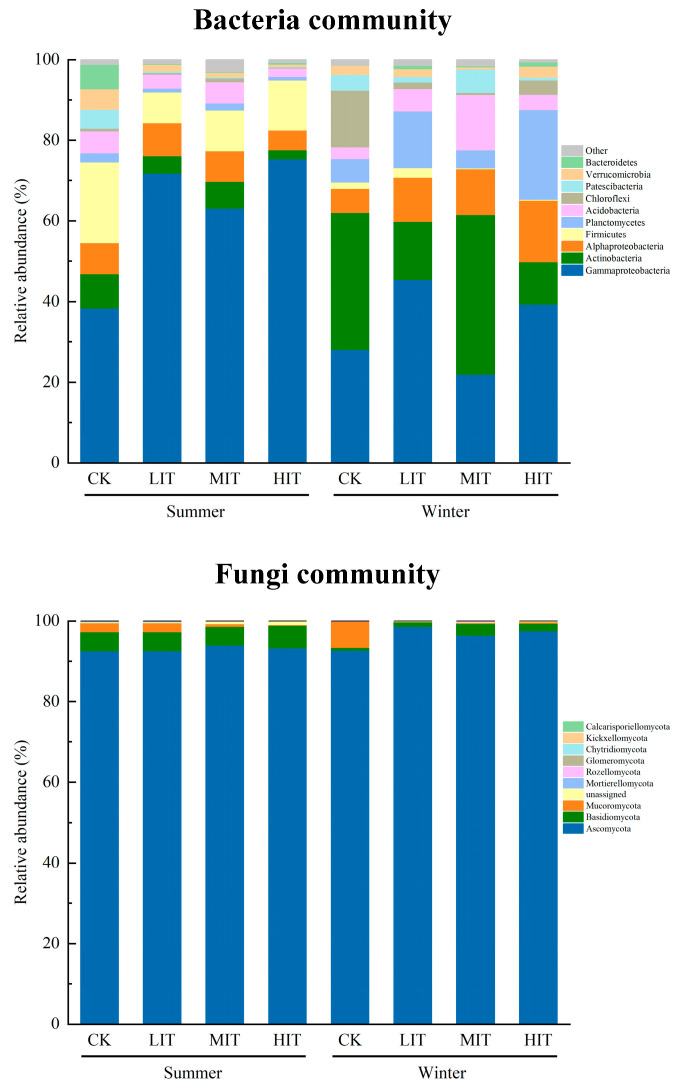
Taxonomic composition of bacteria and fungi communities at phylum level under different thinning intensities. Bacteria phyla with relative abundances below 1% were summarized as ‘other’. CK, control; LIT, low-intensity thinning; MIT, moderate-intensity thinning; HIT, high-intensity thinning.

The microbiome exhibited a specific species composition in summer and winter. The bacterial community was predominantly composed of Gammaproteobacteria (21.95–75.35%), Actinobacteria (2.23–39.60%), Alphaproteobacteria (4.92–15.35%), Firmicutes (0.18–19.99%) and Planctomycetes (0.95–22.25%) (Figure 2 and Table S2). The relative abundances of Gammaproteobacteria (62.14%) and Firmicutes (12.46%) in summer were significantly higher than those in winter (33.72% and 1.07%, respectively). The relative abundances of Actinobacteria (24.59%), Alphaproteobacteria (10.87%), Planctomycetes (11.69%) and Chloroflexi (4.90%) in winter were significantly higher than those in summer (5.42%, 7.12%, 1.50%, and 0.56%, respectively). Thinning significantly increased the relative abundance of Gammaproteobacteria in summer, while significantly reducing the relative abundances of Actinobacteria, Firmicutes, Patescibacteria, and Verrucomicrobia. The relative abundances of Alphaproteobacteria, Planctomycetes, Acidobacteria, and Chloroflexi showed the lowest values in HIT. In winter, with the exception of MIT, thinning significantly increased the relative abundances of Gammaproteobacteria, Planctomycetes, and Bacteroidetes, while significantly decreasing the relative abundance of Actinobacteria. All thinning treatments also demonstrated consistent patterns, with all thinning intensities significantly increasing the relative abundance of Alphaproteobacteria and decreasing the relative abundance of Chloroflexi.

The fungal community was predominantly composed of Ascomycota (92.53–98.63%), Basidiomycota (0.65–5.69%), and Mucoromycota (0.10–6.57%) (Figure 2 and Table S2). The relative abundances of Basidiomycota (4.68%) and Glomeromycota (0.01%) in summer were significantly higher than those in winter (1.67% and 0.001%, respectively). The relative abundance of Ascomycota (96.28%) in winter was significantly higher than that in summer (93.62%). In summer, thinning increased the relative abundance of Ascomycota, although the difference between HIT and CK was not statistically significant. Furthermore, thinning significantly reduced the relative abundances of Mucoromycota, Mortierellomycota, and Glomeromycota. In winter, thinning significantly increased the relative abundance of Ascomycota and significantly decreased the relative abundance of Mucoromycota.

### 3.2. Thinning-Sensitive Microbes

**Figure 3:**
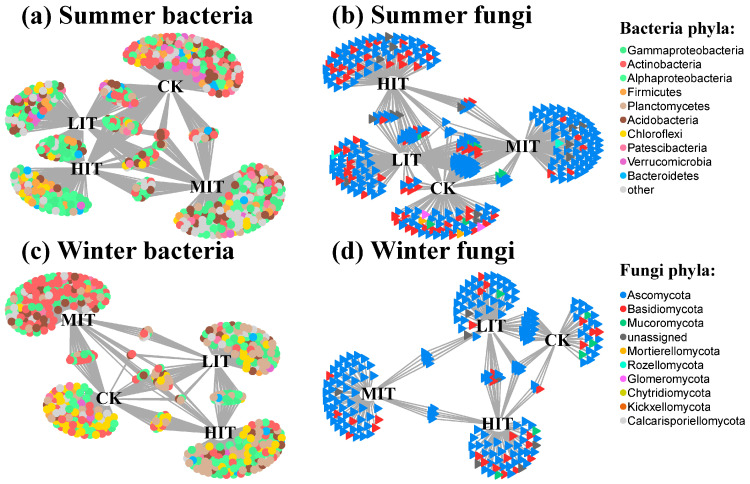
Bipartite networks display thinning-specific bacterial and fungal OTUs in summer and winter using indicator species analysis. CK, control; LIT, low-intensity thinning; MIT, moderate-intensity thinning; HIT, high-intensity thinning.

Using indicator species analysis, we identified individual microorganisms that varied between different thinning intensities, revealing 657 bacterial OTUs and 344 fungal OTUs in summer, and 538 bacterial OTUs and 219 fungal OTUs in winter (Figure S4). Based on indicator species analysis and LRT, we selected tsOTUs and visualized them using a bipartite network (Figure 3). In total, we identified 647 bacterial tsOTUs and 337 fungal tsOTUs, which accounted for 20.28% and 16.51% of the total retained OTUs in summer. Similarly, we identified 532 bacterial tsOTUs and 218 fungal tsOTUs, accounting for 20.28% and 12.62% of the total retained OTUs in winter.

The bipartite network also indicated that thinning led to the differentiation of bacterial and fungal community composition (Figure 3), which was consistent with the results of the previous PCoA and CAP results (Figure 1 and Figure S2). Unique tsOTUs were accumulated within each thinning intensity, while a small number of tsOTUs were shared across all thinning intensities. In winter, the number of bacterial and fungal tsOTUs that were commonly present across different treatments was notably fewer than in summer. Additionally, regardless of the season (summer or winter), the number of shared tsOTUs among fungal communities across treatments was consistently lower than that among bacterial communities. In general, most bacterial tsOTUs belonged to the phyla Actinobacteria, Proteobacteria and Acidobacteria, while most fungal tsOTUs belonged to the phylum Ascomycota (Figures S5 and S6).

### 3.3. Co-Occurrence Networks

Individual-co-occurrence networks were built for bacteria and fungi, respectively (Figure S7). The highest numbers of nodes and edges were detected in the summer bacterial network, followed by the winter bacterial and summer fungal networks. Conversely, the smallest numbers of nodes and edges were detected in the winter fungal network. In terms of average node degree, the winter bacterial network exhibited the highest level of connectivity and complexity, whereas the winter fungal network exhibited the lowest level of connectivity and complexity.

**Figure 4:**
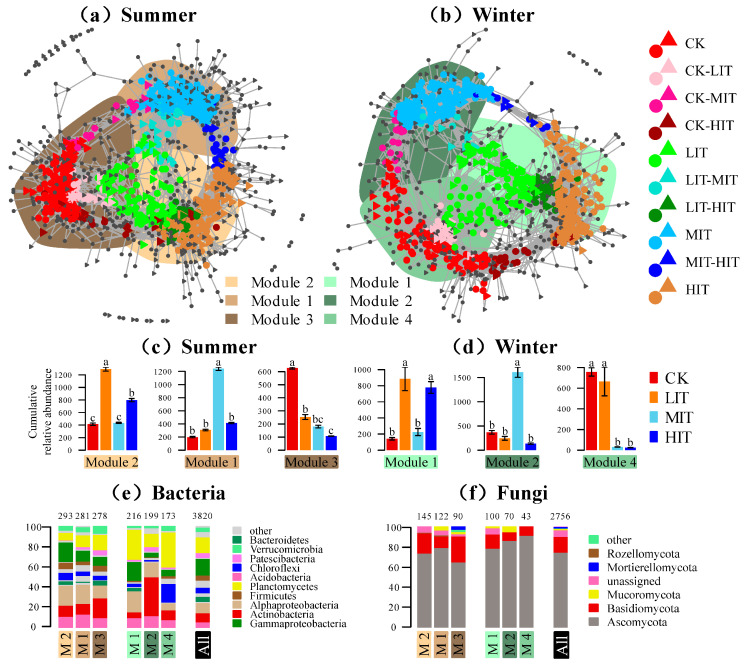
Co-occurrence patterns of thinning-sensitive OTUs. (**a**) Co-occurrence patterns of thinning-sensitive bacterial and fungal OTUs in summer. (**b**) Co-occurrence patterns of thinning-sensitive bacterial and fungal OTUs in winter. Circles indicate bacteria, triangles indicate fungi, and asterisks indicate keystone OTUs. (**c**,**d**) Cumulative relative abundance counts per million (y-axis in ×1000). Different lowercase letters indicate significant differences (*p* < 0.05). (**e**,**f**) Composition of the top 3 modules in summer and winter networks. Bacteria and fungi phyla with relative abundances below 1% in the entire dataset were summarized as “other”. The module order is sorted by the number of thinning-sensitive OTUs in the module. The numbers on the stacked bar chart represent the total number of OTUs in the module. CK, control; LIT, low-intensity thinning; MIT, moderate-intensity thinning; HIT, high-intensity thinning; M1, module 1; M2, module 2; M3, module 3; M4, module 4; All, the overall taxonomic distribution in the entire dataset.

We constructed an overall network of bacterial and fungal communities in summer and winter (Figure 4 and Table 3). The rhizosphere microbial network was more complex in summer than in winter. The network comprised 1459 (988 bacterial and 471 fungal) and 1117 (810 bacterial and 307 fungal) nodes in summer and winter, respectively. The mean node degrees were 18 and 18.08%, respectively. The interactions among bacteria were the strongest (6415 and 5769 in summer and winter, respectively), followed by the interactions between bacteria and fungi (5132 and 3343), while the interactions among fungi were the weakest (1581 and 985). In summer, compared to CK (203), LIT (115) and HIT (150) reduced the number of nodes, while MIT (227) increased the number of nodes. In winter, compared to CK (113), all thinning intensities increased the number of nodes, with LIT, MIT, and HIT having 129, 190, and 170 nodes, respectively. Additionally, a certain number of tsOTUs were shared among different thinning intensities, mirroring the results of the previous bipartite networks. For example, the co-occurrence networks showed that CK and LIT, LIT, and HIT shared a large number of tsOTUs, which was also reflected in the bipartite network. Similarly, the impact of seasons was also presented in the co-occurrence networks. In summer, LIT and MIT shared 38 tsOTUs, while in winter, there were only 14, which was consistent with the bipartite network.

Based on the identification of the top 20 most abundant modules in each season, we highlighted and coded top 3 modules as Module 2 (M2), Module 1 (M1), and Module 3 (M3) in summer, and as Module 1 (M1), Module 2 (M2), and Module 4 (M4) in winter (Figure 4a,b and Figure S8). All the modules showed strong responses to thinning and exhibited independence from each other in the networks, revealing the driving processes of community differences. For example, M2, M1, and M3 in summer contained tsOTUs specific to LIT, MIT, and HIT, respectively. Similarly, in winter, numerous tsOTUs assigned to LIT and HIT were predominantly located in M1, tsOTUs assigned to MIT were located in M2, and tsOTUs assigned to CK were located in M4, with a small number of tsOTUs assigned to LIT also clustering in M4. The species composition of these modules covered a wide range of bacterial and fungal phyla that differed from each other (Figure 4c,d). The major bacterial phyla were Gammaproteobacteria, Actinobacteria, Alphaproteobacteria, Planctomycetes, and Chloroflexi, which accounted for approximately half of all taxa. Among fungi, Ascomycota was dominant to a great extent, and its dominance was even higher in winter compared to that in summer.

### 3.4. Keystone Taxa in Meta Co-Occurrence Networks

**Figure 5:**
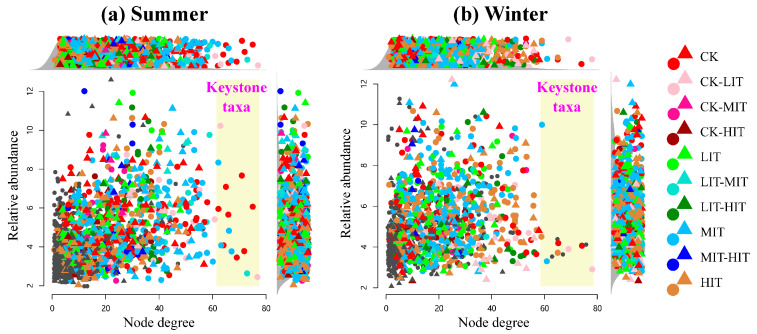
Degree of co-occurrence and abundances of the thinning-sensitive OTUs in summer and winter. Circles and triangles represent bacteria and fungi, respectively. The yellow shaded parts represent keystone taxa. CK, control; LIT, low-intensity thinning; MIT, moderate-intensity thinning; HIT, high-intensity thinning.

In the co-occurrence networks of summer and winter, 15 and 12 keystone OTUs were identified, respectively, all of which were bacteria (Figure 5, Figures S9 and S10 and Table S3). The keystone OTUs in summer belonged to the phyla Acidobacteria, Actinobacteria, Firmicutes, Planctomycetes, Nitrospirae, Planctomycetes, Proteobacteria, and Verrucomicrobia. The keystone OTUs in winter belonged to the phyla Acidobacteria, Actinobacteria, Chloroflexi, and Planctomycetes.

In summer, keystone OTUs were all sensitive to thinning, and the relative abundances of most OTUs decreased after thinning (Figure S9 and Table S3). Notably, MIT enriched some keystone OTUs, such as bOtu001865, bOtu001091, bOtu001095, bOtu001467, and bOtu000063. In winter, eight keystone OTUs were sensitive to thinning, and the relative abundances of some OTUs also decreased after thinning (Figure S10 and Table S3). bOtu001439 and bOtu000037 showed high abundance in MIT, while bOtu001855 and bOtu000704 showed high abundance in HIT.

## 4. Discussion

### 4.1. Effects of Thinning on Microbial Diversities and Compositions

In this study, the key factor leading to the change of α-diversity was season, and thinning had no significant effects on α-diversity indices, which might be related to the dynamic adaptation of rhizosphere microbial community to interference under the participation of plant stress response [40]. The microbial communities in bulk soil often showed regular responses to thinning. For example, Liu et al. [41] found that with increasing thinning intensity, the α-diversity indices of soil bacteria and fungi in *Cryptomeria japonica* var. *sinensis* plantations showed a trend of first decreasing and then increasing. In contrast, a study on Chinese fir plantations reached an opposite conclusion, where the soil microbial Shannon indices showed a trend of first increasing and then decreasing in most seasons, regardless of whether it was measured in the 0–10 cm soil layer or the 10–25 cm soil layer [42]. However, there have emerged reports indicating the absence of notable alterations in the bacterial and fungal α-diversity indices, despite variations in thinning intensity [15,43]. These discrepancies might be related to differences in thinning time, forest type, forest age, stand types, and understory vegetation diversity. Unlike bulk soil, the rhizosphere environment is strongly influenced by root exudates. Thinning that disturbs the growth of individual trees may affect the composition of root exudates, thereby altering the structure of carbon sources available to microorganisms [44]. Therefore, under thinning measures, the diversity of rhizosphere microbial communities, in comparison to soil microbial communities, emerges as a complex outcome driven by multiple factors, which potentially account for the irregular variations observed in microbial community diversity within this study. Given the current limited research, a comprehensive interpretation necessitates the integration of root exudate measurements in future studies. Nonetheless, the β-diversity analysis based on Bray–Curtis distance still indicated that thinning significantly influenced the microbial community structure (Figure 1).

Thinning also led to the enrichment of specific microbial taxa. Gammaproteobacteria are often referred to as “opportunists” with a broad range of substrate utilization capabilities, enabling them to respond rapidly to changes in the surrounding environment [45]. In this study, the relative abundance of Gammaproteobacteria was significantly higher in summer compared to that in winter, and also significantly higher in thinning treatments compared to CK, which might be closely related to the abundant exogenous carbon in the rhizosphere caused by the improved growth conditions of trees (Table S2). Actinobacteria are eutrophic microorganisms involved in the decomposition of stable macromolecular compounds, such as lignin and cellulose [46]. The high abundance of Actinobacteria observed in winter is intimately related to the increased input of rhizosphere litter, particularly fine-root necromass [10]. Meanwhile, regardless of whether it was summer or winter, thinning consistently reduced the relative abundance of Actinobacteria. A plausible explanation for this phenomenon is that the increased allocation of nutrients to the root system by trees prolongs the lifespan of fine roots and decreases their turnover rate, leading to a reduction in fine-root necromass [47,48]. Ascomycota dominated the fungal community with a remarkably high relative abundance (ranging from 92.53% to 98.63%). This phenomenon may be related to the rhizosphere sampling methodology employed in this study, which involved removing the tertiary fine roots followed by laboratory extraction of rhizosphere microorganisms [39]. The fine roots of *P. massoniana* extensively form symbiotic relationships with ectomycorrhizal fungi, which predominantly belong to Ascomycota. This specific symbiosis likely contributed to the observed dominance of Ascomycota in the sequencing results [7]. Consistent with previous studies, this research found that thinning can significantly increase the abundance of Ascomycota, which is associated with the improved growth space for ectomycorrhizal fungi and the enrichment of exogenous carbon in the rhizosphere following thinning interventions [15,41].

### 4.2. Effects of Thinning on Microbial Co-Occurrence Network

The tsOTUs were identified through indicator species analysis. Each thinning intensity supported a specialized microbial subset, and some tsOTUs were shared among different thinning intensities. Our previous study on this plantation found that thinning affected the aggregation of rhizosphere tsOTUs by driving alterations in understory vegetation [7]. Furthermore, the number of tsOTUs in summer was higher than that in winter, suggesting that ignoring seasonal factors would lead to an incomplete understanding of the impact of thinning on rhizosphere microbial communities.

The bacterial tsOTUs primarily belonged to Proteobacteria, Actinobacteria, and Acidobacteria. These bacterial taxa have been reported to be abundant in acidic forest topsoil and produce carbohydrate-active enzymes that enable their access to carbon in cellulose or hemicellulose [49]. Consistent with the relative abundance patterns of species, fungal tsOTUs were primarily affiliated with Ascomycota. In addition, a considerable number of tsOTUs belonged to extremely-low-abundance bacterial taxa, such as Gemmatimonadetes, Chlamydiae, and Armatimonadetes. This indicates that, under thinning measures, rare bacteria also play an important role in shaping the rhizosphere microbial communities.

Thinning changed the pattern of the co-occurrence network (Figure 4). Whether in summer or winter, MIT had the highest number of nodes, indicating that MIT increased the complexity of the rhizosphere network to a certain extent. Previous studies have suggested that network complexity is related to resource availability, such as soil water, carbon, and nutrients [8,50]. Similarly, in this study, MIT showed the highest soil water content, soil organic carbon content, and the lowest pH value in both summer and winter, which might lead to increased complexity of the microbial network (Table 3). We identified the top three modules of the networks and found that the distribution patterns of tsOTUs within these modules showed a relatively consistent trend between summer and winter. (Figure 4c,d and Figure S8). This finding indicated a high adaptability of the rhizosphere microbial network structure to seasonal variations. In these modules we also found that the rhizosphere microorganisms had different degrees of adaptation to different thinning intensities (Figure 4e,f). For example, in summer M2 module and winter M1 module, which were enriched with OTUs sensitive to LIT and HIT, the relative abundances of Alphaproteobacteria and Gammaproteobacteria were higher than those in other modules. In contrast, in the summer M1 module and winter M2 module, which were enriched with OTUs sensitive to MIT, the relative abundance of Acidobacteria was higher than that in other modules. However, the specific adaptation mechanism of rhizosphere microorganisms to thinning requires a detailed elaboration that integrates considerations of root exudates and substrate concentration [44].

Keystone taxa play a critical role in shaping microbial community structure and function, serving as indicative markers for responding to changes in the external environment [51,52]. In this study, the 15 and 12 keystone OTUs identified in summer and winter, respectively, were all bacteria, indicating that bacteria play a more significant role than fungi in maintaining the structural integrity of rhizosphere microbial networks. This finding is consistent with previous research conclusions on thinning [8]. All the summer keystone OTUs belonged to tsOTUs, and the relative abundances of most of the keystone OTUs (10 OTUs) showed a declining trend after thinning, demonstrating that thinning can lead to a decrease in the connectivity of the rhizosphere microbial network (Figure S9 and Table S3). It is noteworthy that the relative abundances of five keystone OTUs peaked under MIT treatment, which may also be attributed to the enhanced resource availability resulting from the MIT. Notably, one of these OTUs belongs to the Nitrospirae, a bacterial group well-established as a crucial player in the nitrogen cycle, thereby reinforcing our hypothesis [53]. Compared to summer, the composition of winter keystone OTUs might be subject to more complex influences due to plant dormancy, with the emergence of four OTUs that were insensitive to thinning. Among the eight keystone OTUs belonging to tsOTUs, two keystone OTUs showed maximum values under the HIT treatment, while another two showed maximum values under the MIT treatment. Overall, similar to the trend observed in summer, the relative abundances of most keystone OTUs decreased after thinning. It can be inferred from these studies that the assembly process of rhizosphere microorganisms is jointly driven by rhizosphere activity and root exudation chemistry under the influence of seasons. However, due to the extremely limited research, the functions of key species in this study remained poorly understood, and further research is required to clarify the regulatory mechanisms of microbial ecological processes.

## 5. Conclusions

This study preliminarily explored the response of rhizosphere microbial communities to thinning in summer and winter, and we found that the characteristics of rhizosphere microbial communities were complex results driven by multiple factors. Despite the lack of discernible patterns in changes to α-diversity, thinning significantly shaped the rhizosphere microbial community structure and led to the accumulation of specific microbial taxa. Thinning also caused specific responses from the microbial meta-co-occurrence network modules, with the network complexity being the highest in the MIT treatment. Similarly, while network connectivity exhibited a declining trend after thinning, MIT facilitated the enrichment of certain key taxa. It is plausible that these observations are underpinned by the increased availability of soil resources resulting from the MIT treatment. Consequently, we infer that moderate thinning intensity could potentially represent the optimal thinning intensity for the studied *P. massoniana* plantations. Furthermore, we also identified season as a non-negligible factor in this study. Although winter did not alter the modular structure of the network, the changes in network connectivity it induced indirectly corroborated that the assembly process of rhizosphere microorganisms is driven by complex mechanisms under the influence of seasonal variations. Future research should further focus on the functional traits of thinning-sensitive keystone taxa, as well as rhizosphere dynamics and rhizodeposition characteristics, in order to provide enhanced support for the development of sustainable forestry management practices.

## Figures and Tables

**Table 1:** Stand characteristics of the experimental plots (mean ± SD).

Characteristics	Summer				Winter			
	CK	LIT	MIT	HIT	CK	LIT	MIT	HIT
Stand density (tree 600 m^−2^)	92	81	63	45	/	/	/	/
Diamater at breast height (cm)	16.1 ± 2.0	16.8 ± 1.6	17.5 ± 2.2	18.2 ± 1.2	/	/	/	/
Soil water content (mg g^−1^)	309.21 ± 45.88	238.63 ± 20.15	364.34 ± 82.92	362.00 ± 63.50	245.33 ± 56.57	289.68 ± 13.10	304.51 ± 53.08	257.10 ± 79.40
Soil bulk density (g cm^−3^)	1.34 ± 0.14	1.52 ± 0.05	1.24 ± 0.04	1.28 ± 0.21	1.17 ± 0.09	1.37 ± 0.13	1.24 ± 0.18	1.29 ± 0.10
pH	4.98 ± 0.02	4.88 ± 0.01	4.47 ± 0.03	4.87 ± 0.04	4.73 ± 0.02	4.93 ± 0.02	4.64 ± 0.05	4.96 ± 0.03
Soil temperature (°C)	23.73 ± 0.06	23.77 ± 0.06	24.47 ± 0.06	24.43 ± 0.06	10.83 ± 0.06	11.13 ± 0.06	11.20 ± 0.10	11.60 ± 0.10
Soil organic carbon (g kg^−1^)	8.40 ± 0.37	12.39 ± 0.69	16.57 ± 6.22	9.97 ± 0.69	6.73 ± 1.83	6.85 ± 0.33	8.83 ± 0.32	4.97 ± 0.83
Total nitrogen (g kg^−1^)	0.78 ± 0.05	1.26 ± 0.18	0.85 ± 0.07	0.68 ± 0.03	0.98 ± 0.01	0.75 ± 0.03	0.83 ± 0.04	0.58 ± 0.04
Total phosphorus (g kg^−1^)	0.10 ± 0.05	0.11 ± 0.01	0.12 ± 0.04	0.11 ± 0.06	0.08 ± 0.05	0.06 ± 0.01	0.10 ± 0.02	0.11 ± 0.05
Total potassium (g kg^−1^)	21.10 ± 4.47	19.90 ± 0.86	16.51 ± 2.01	16.79 ± 0.87	15.64 ± 0.42	16.63 ± 1.51	22.34 ± 6.24	17.04 ± 1.41

CK, control; LIT, low-intensity thinning; MIT, moderate-intensity thinning; HIT, high-intensity thinning.

**Table 2:** Results of Kruskal–Wallis test for α-diversity indices of rhizosphere microbial communities. (***: *p* < 0.001).

Group	α-Diversity Index	*H*	*p*
Season	Bacterial Chao index	11.603	<0.001 ***
	Bacterial Pielou index	12.813	<0.001 ***
	Fungi Chao index	11.213	<0.001 ***
	Fungi Pielou index	0.030	0.862
Thinning intensity	Bacterial Chao index	5.913	0.116
	Bacterial Pielou index	3.233	0.357
	Fungi Chao index	4.540	0.209
	Fungi Pielou index	0.613	0.893

**Table 3:** Properties of summer and winter meta co-occurrence networks.

Season	Number of Nodes		Number of Edges			Average Connectivity	Number of tsOTUs		Number of Keystones OTUs	
	B	F	B-B	F-F	B-F		B	F	B	F
Summer	988	471	6415	1581	5132	18.00	645	336	15	0
Winter	810	307	5769	985	3343	18.08	531	218	12	0

B, bacteria; F, fungi; B-B, bacterium–bacterium interaction; F-F, fungus–fungus interaction; B-F, bacterium–fungus interaction.

## Data Availability

The original sequencing data for this study is publicly available. These data can be found here: NCBI, PRJNA1248207 (https://www.ncbi.nlm.nih.gov/bioproject/1248207) (accessed on 9 April 2025).

## Supplementary Materials

The following supporting information can be downloaded at: https://www.mdpi.com/article/10.3390/microorganisms13061357/s1, Figure S1: Alpha diversity of bacteria (a,b) and fungi (c,d) communities under different thinning intensities; Figure S2: Principal coordinates analysis (PCoA) of bacteria and fungi in winter and summer based on Bray–Curtis distance; Figure S3: Specific sets of bacteria and fungi in summer and winter; Figure S4: Venn diagrams display the number of OTUs responding to thinning by using indicator species analysis (orange) and edgeR (blue); Figure S5: Relative abundances of thinning-sensitive bacterial and fungal OTUs at phylum level; Figure S6: Relative abundances of thinning-sensitive bacterial and fungal OTUs; Figure S7: Individual co-occurrence networks of summer and winter microorganisms under different thinning intensities; Figure S8: Number of OTUs in the top 20 modules for meta co-occurrence networks; Figure S9: Relative abundances (counts per million, CPM) of keystone bOTUs in summer; Figure S10: Relative abundances (counts per million, CPM) of keystone bOTUs in winter; Table S1: Results of PERMANOVA testing the effects of TI and Season on bacterial and fungal communities; Table S2: Results of multiple comparisons tests for the effects of thinning and season on rhizosphere microbial community composition at phylum level; Table S3: Description of keystone OTUs identified in summer and winter. References [54,55,56,57] are cited in the Supplementary Materials.
