# Supplementary material for: Early Response of Rhizosphere Microbial Community Network Characteristics to Thinning Intensity in Pinus massoniana Plantations
Source: Microorganisms. 2025 Jun 11;13(6):1357. doi: 10.3390/microorganisms13061357 (PMC12195532; doi:10.3390/microorganisms13061357)
Supplement: Supplementary file 1 [file microorganisms-13-01357-s001.zip › microorganisms-3661921-supplementary.pdf]

SUPPLEMENTARY FIGURES

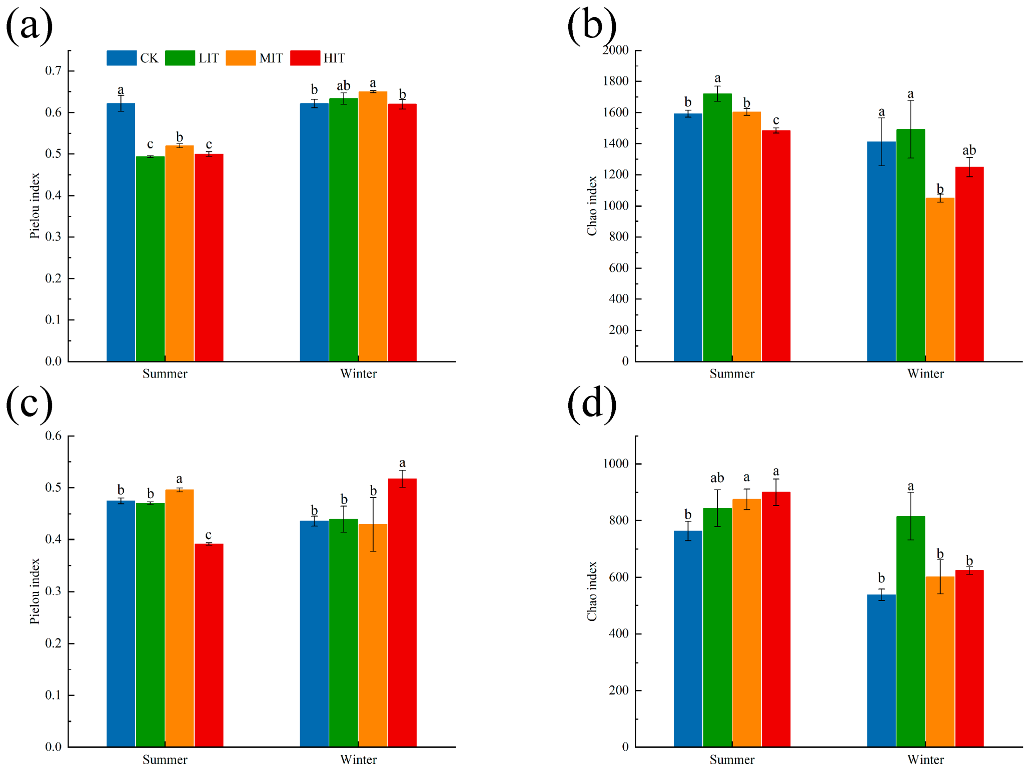

Figure S1. Alpha diversity of bacteria (a-b) and fungi (c-d) communities under different thinning intensities. Different lowercase letters represent significant differences among different thinning intensities within the same season. CK, control; LIT, low-intensity thinning; MIT, moderate-intensity thinning; HIT, high-intensity thinning.

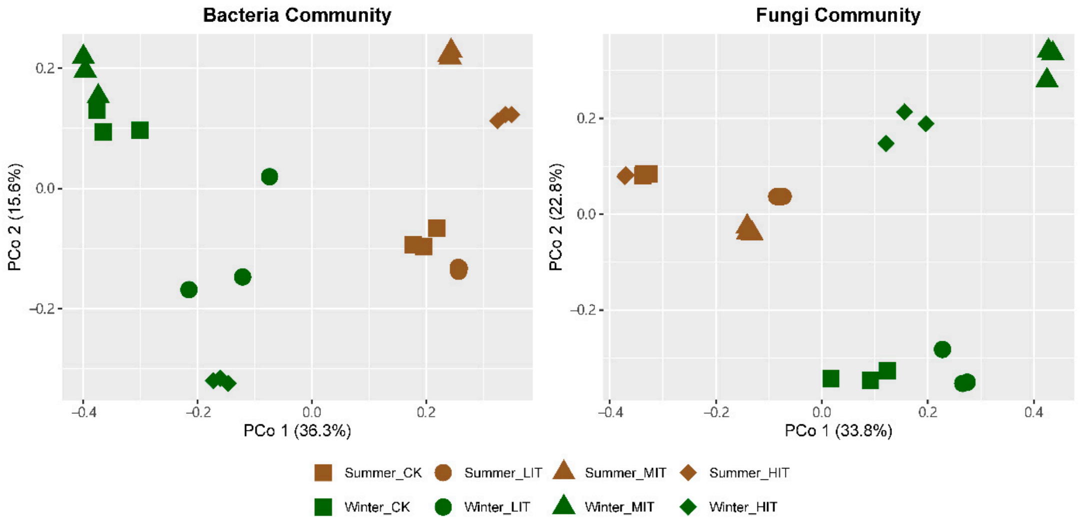

Figure S2. Principal coordinates analysis (PCoA) of bacteria and fungi in winter and summer based on Bray-Curtis distance. CK, control; LIT, low-intensity thinning; MIT, moderate-intensity thinning; HIT, high-intensity thinning. The same below.

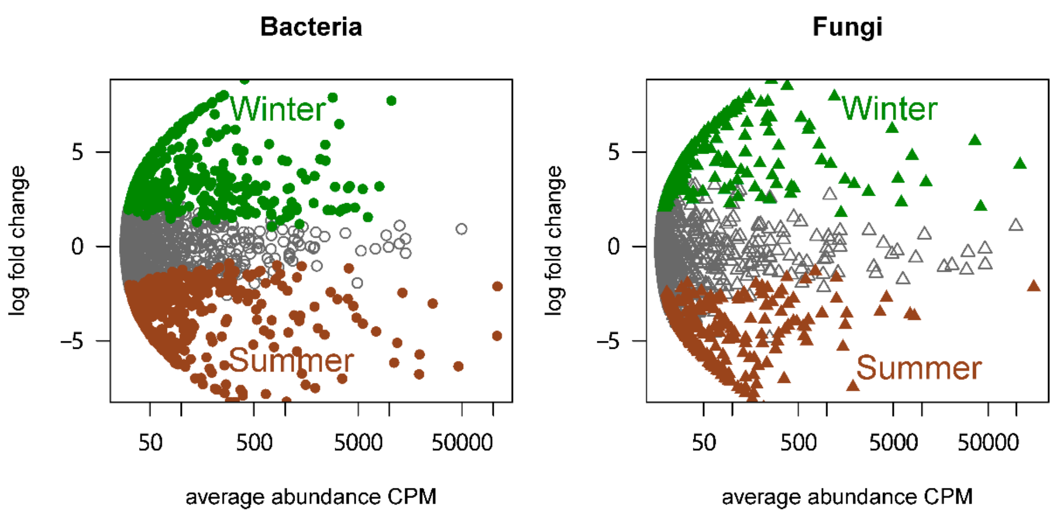

Figure S3. Specific sets of bacteria and fungi in summer and winter. X-axis represents average OTU abundance (counts per million, CPM), and Y-axis represents log-fold change (winter relative to summer). winter and summer-specific OTUs were colored in green and brown, respectively, and non-differentially abundant OTUs are in gray (likelihood ratio test, $P < 0.05$).

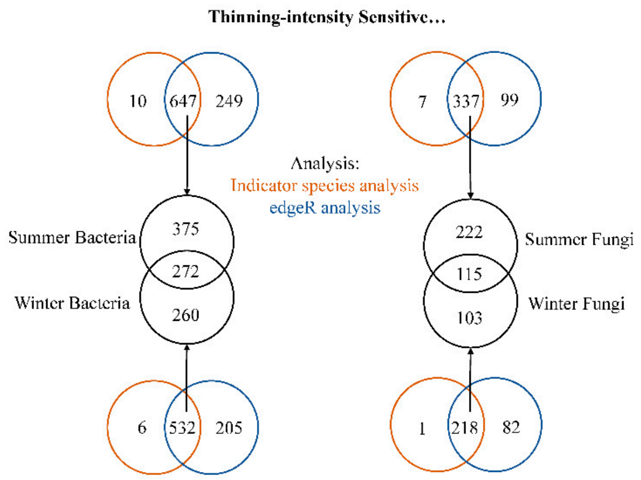

Figure S4. Venn diagrams display the number of OTUs responding to thinning by using indicator species analysis (orange) and edgeR (blue). OTUs supported by both methods were defined as thinning-sensitive OTUs (tsOTUs).

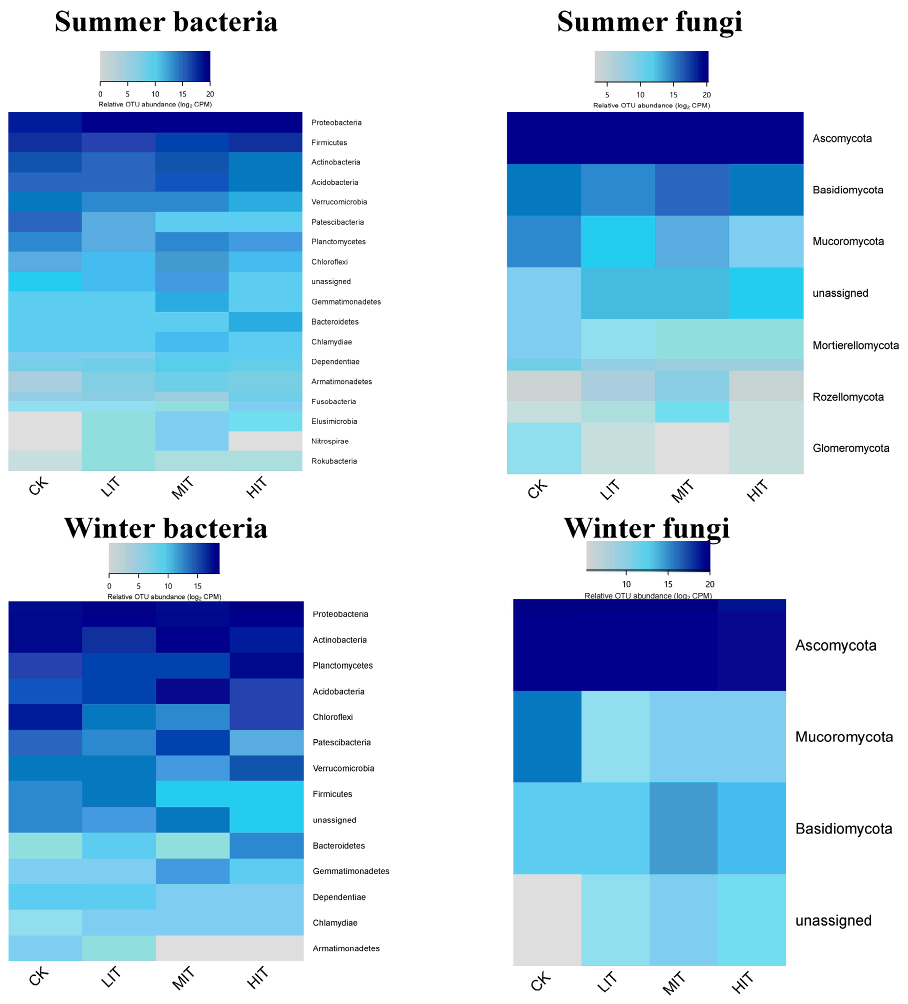

Figure S5. Relative abundances of thinning-sensitive bacterial and fungal OTUs at phylum level.

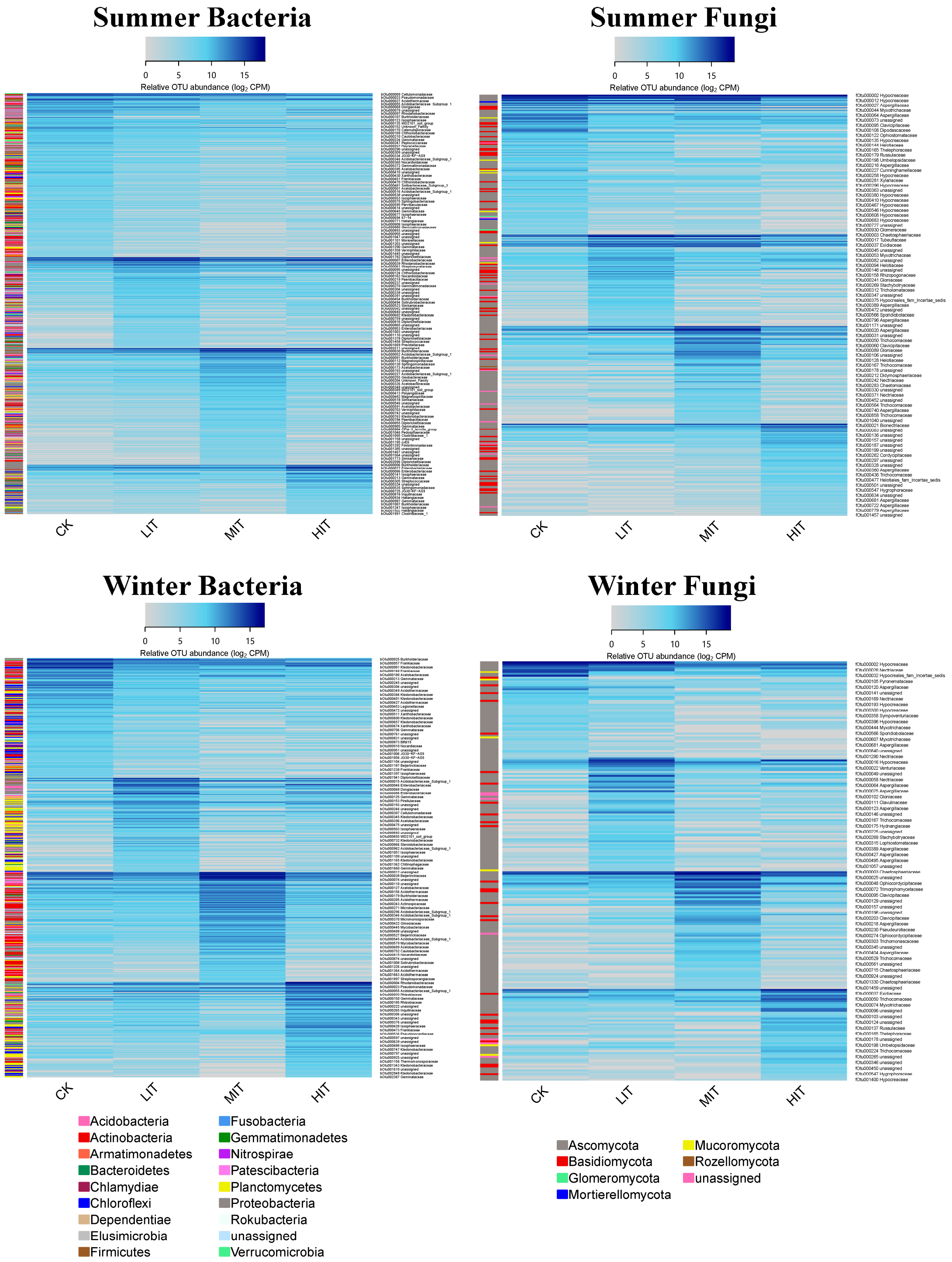

Figure S6. Relative abundances of thinning-sensitive bacterial and fungal OTUs. OTUs are labeled with the phylum level taxonomy assignment indicated by the colored bars.

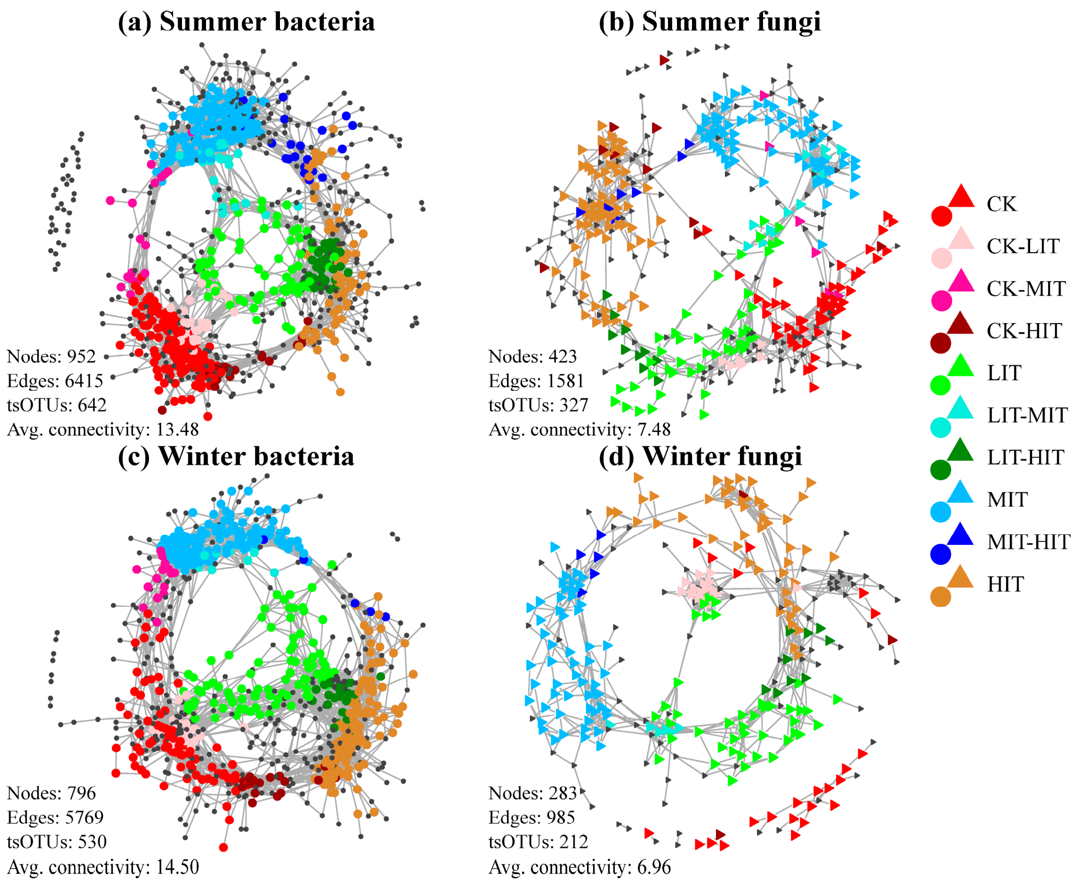

Figure S7. Individual cooccurrence networks of summer and winter microorganisms under different thinning intensities. Circles and triangles represent bacteria and fungi OTUs, respectively. The numbers of nodes, edges and tsOTUs and average connectivity were listed under each network. tsOTUS, thinning-sensitive OTUs.

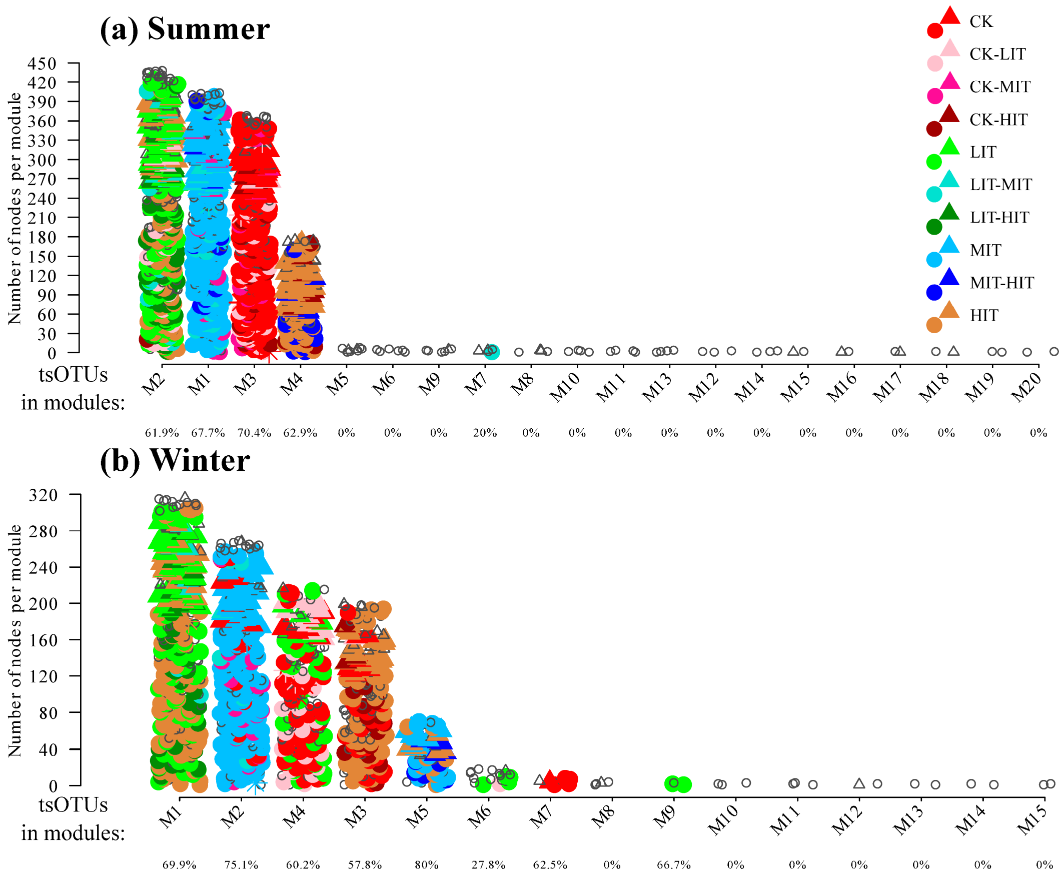

Figure S8. Number of OTUs in the top 20 modules for meta cooccurrence networks. Circle and triangle represent bacteria and fungi, respectively. Percentage is the proportion of thinning-sensitive OTUs to total OTUs in each module.

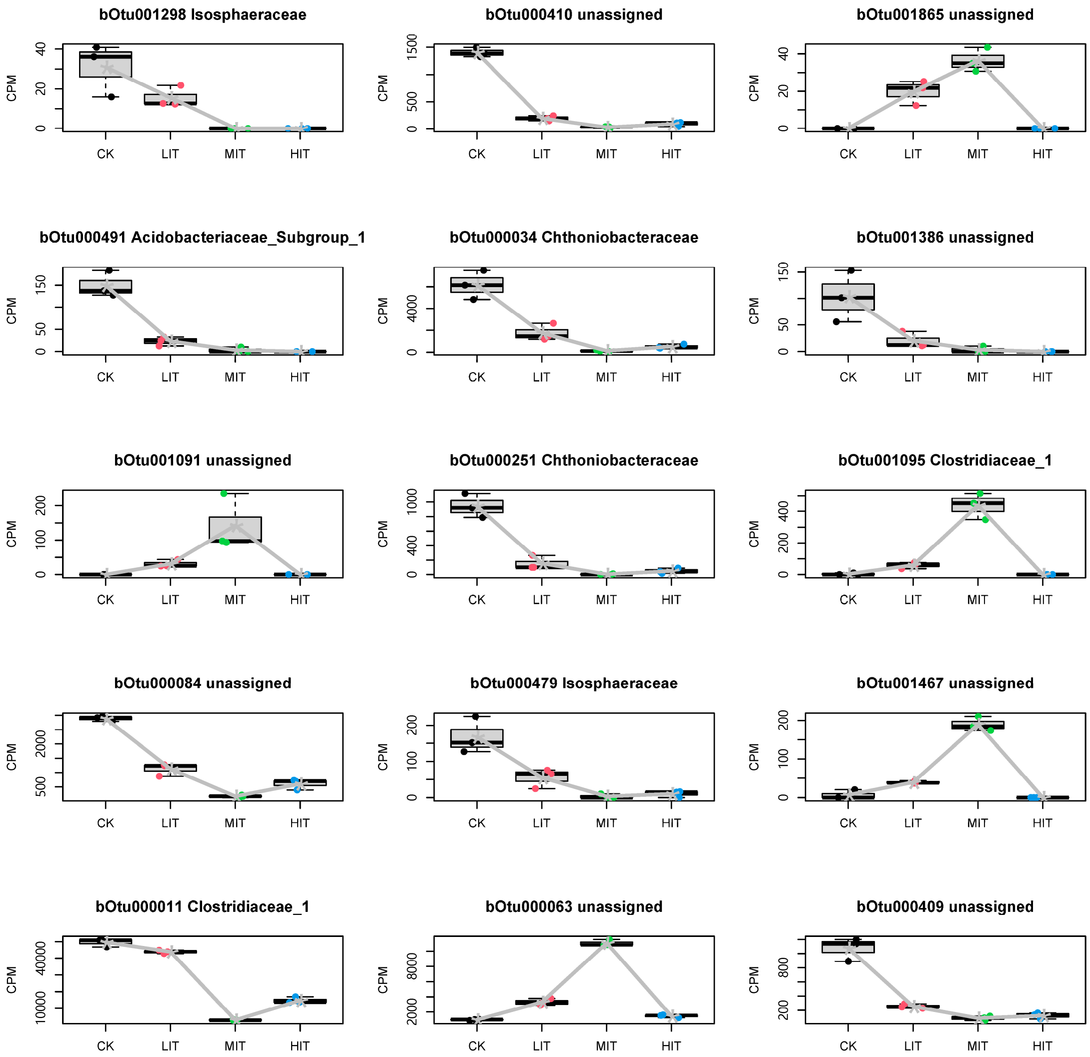

Figure S9. Relative abundances (counts per million, CPM) of keystone bOTUs in summer. Means within each thinning intensity are indicated in gray stars.

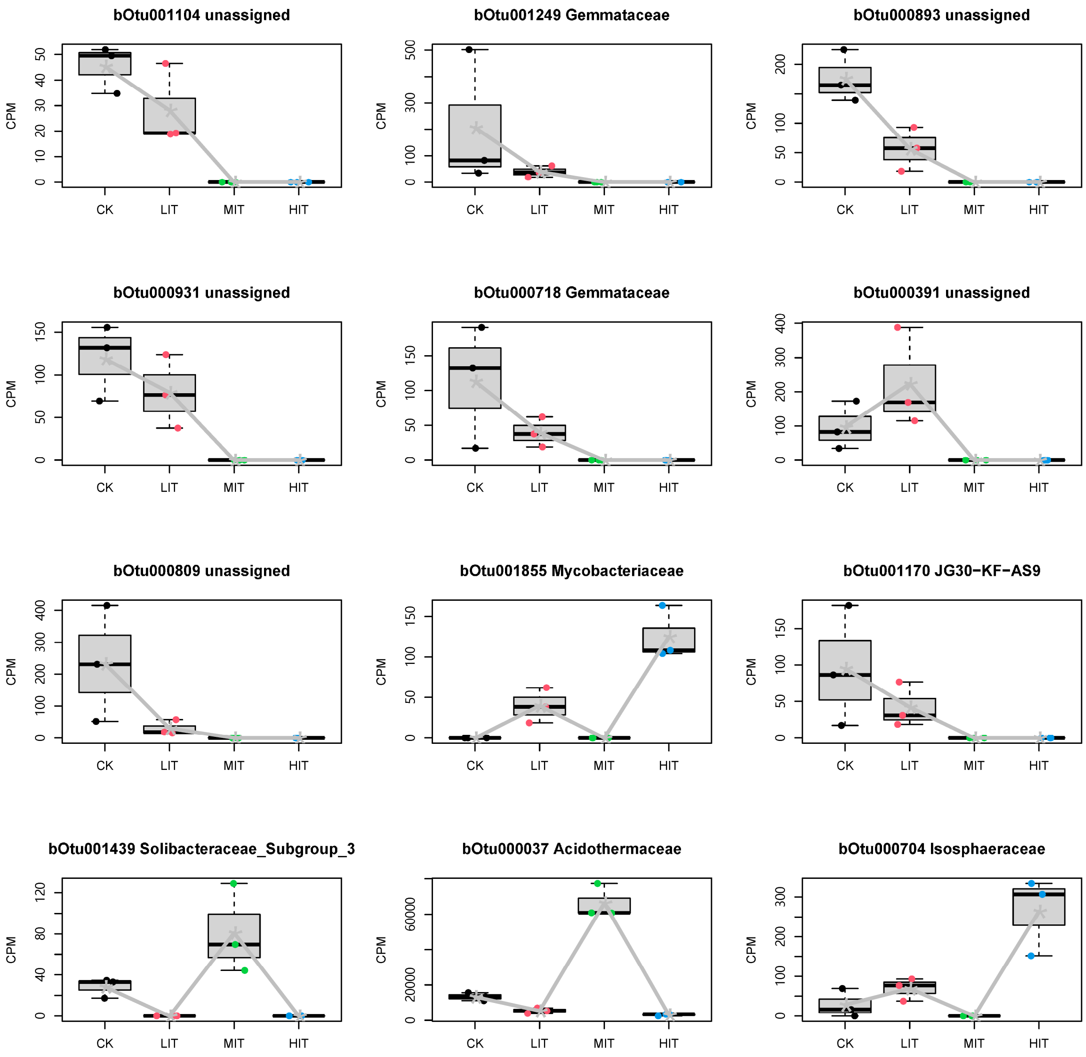

Figure S10. Relative abundances (counts per million, CPM) of keystone bOTUs in winter. Means within each thinning intensity are indicated in gray stars.

SUPPLEMENTARY TABLES

Table S1. Results of PERMANOVA testing the effects of TI and Season on bacterial and fungal communities. (***: $P < 0.001$)

	Bacteria		Fungi	
	R^2	F	R^2	F
TI	0.293	19.368^{***}	0.313	27.569^{***}
Season	0.335	66.474^{***}	0.288	75.901^{***}
TI*Season	0.292	19.291^{***}	0.338	29.742^{***}

Table S2. Results of multiple comparisons tests for the effects of thinning and season on rhizosphere microbial community composition at phylum level.

	Summer				Winter				Summer	Winter
	CK	LIT	MIT	HIT	CK	LIT	MIT	HIT		
Bacteria community at Phylum level										
Gammaproteobacteria	c	a	b	a	b	a	b	a	A	B
Actinobacteria	a	c	b	d	a	b	a	b	B	A
Alphaproteobacteria	a	a	a	b	c	b	b	a	B	A
Firmicutes	a	d	c	b	a	a	a	a	A	B
Planctomycetes	a	b	ab	b	c	b	c	a	B	A
Acidobacteria	a	b	a	c	c	b	a	c	A	A
Chloroflexi	b	c	a	c	a	b	b	b	B	A
Patescibacteria	a	b	b	b	ab	bc	a	c	A	A
Verrucomicrobia	a	b	b	b	a	a	a	a	A	A
Bacteroidetes	a	a	a	a	b	a	b	a	A	A
other	b	b	a	c	a	a	a	b	A	A
Fungi community at Phylum level										
Ascomycota	c	a	ab	bc	b	a	a	a	B	A
Basidiomycota	b	c	b	a	a	a	a	a	A	B
Mucoromycota	a	b	b	c	a	b	b	b	A	A
unassigned	c	a	b	a	a	a	a	a	A	B
Mortierellomycota	a	b	b	b	a	a	a	a	A	A
Rozellomycota	b	b	a	b	b	b	a	b	A	A
Glomeromycota	a	b	b	b	a	a	a	a	A	B
Chytridiomycota	a	a	a	a	a	a	a	a	A	A
Kickxellomycota	/	/	/	/	/	/	/	/	/	/
Calcarisporiellomycota	/	/	/	/	/	/	/	/	/	/

Different lowercase letters represent significant differences among different thinning intensities within the same season, and different capital letters represent significant differences between summer and winter. CK, control; LIT, low-intensity thinning; MIT, moderate-intensity thinning; HIT, high-intensity thinning.

Table S3. Description of keystone OTUs identified in summer and winter

	Phylum	Class	Order	Family	Genus	Node	Degree	tsOTU
Summer								
Bacteria	Planctomycetes	Planctomycetacia	Isosphaerales	Isosphaeraceae	unassigned	bOtu001298	77	Yes
	Actinobacteria	Thermoleophilia	Gaiellales	unassigned	unassigned	bOtu000410	75	Yes
	Acidobacteria	Acidobacteriia	Acidobacteriales	unassigned	unassigned	bOtu001865	73	Yes
	Acidobacteria	Acidobacteriia	Acidobacteriales	Acidobacteriaceae_Subgroup_1	Granulicella	bOtu000491	72	Yes
	Verrucomicrobia	Verrucomicrobiae	Chthoniobacterales	Chthoniobacteraceae	Candidatus_Udaeobacter	bOtu000034	71	Yes
	Actinobacteria	Thermoleophilia	Gaiellales	unassigned	unassigned	bOtu001386	69	Yes
	Nitrospirae	Thermodesulfovibrionia	unassigned	unassigned	unassigned	bOtu001091	67	Yes
	Verrucomicrobia	Verrucomicrobiae	Chthoniobacterales	Chthoniobacteraceae	Candidatus_Udaeobacter	bOtu000251	66	Yes
	Firmicutes	Clostridia	Clostridiales	Clostridiaceae_1	unassigned	bOtu001095	66	Yes
	Proteobacteria	Alphaproteobacteria	unassigned	unassigned	unassigned	bOtu000084	65	Yes
	Planctomycetes	Planctomycetacia	Isosphaerales	Isosphaeraceae	unassigned	bOtu000479	64	Yes
	Proteobacteria	Alphaproteobacteria	Rickettsiales	unassigned	unassigned	bOtu001467	63	Yes
	Firmicutes	Clostridia	Clostridiales	Clostridiaceae_1	Clostridium_sensu_stricto_10	bOtu000011	63	Yes
	Acidobacteria	Acidobacteriia	Acidobacteriales	unassigned	unassigned	bOtu000063	62	Yes
	Actinobacteria	Thermoleophilia	Gaiellales	unassigned	unassigned	bOtu000409	62	Yes
Winter								
	Acidobacteria	Subgroup_5	unassigned	unassigned	unassigned	bOtu001104	78	Yes
	Planctomycetes	Planctomycetacia	Gemmatales	Gemmataceae	Fimbriiglobus	bOtu001249	76	No
	Acidobacteria	Subgroup_5	unassigned	unassigned	unassigned	bOtu000893	74	Yes
	Planctomycetes	BD7-11	unassigned	unassigned	unassigned	bOtu000931	69	Yes
	Planctomycetes	Planctomycetacia	Gemmatales	Gemmataceae	unassigned	bOtu000718	67	No
	Acidobacteria	Holophagae	Subgroup_7	unassigned	unassigned	bOtu000391	65	No

	Armatimonadetes	Chthonomonadetes	Chthonomonadales	unassigned	unassigned	bOtu000809	65	Yes
	Actinobacteria	Actinobacteria	Corynebacteriales	Mycobacteriaceae	Mycobacterium	bOtu001855	65	Yes
	Chloroflexi	Ktedonobacteria	Ktedonobacterales	JG30-KF-AS9	unassigned	bOtu001170	63	No
	Acidobacteria	Acidobacteriia	Solibacterales	Solibacteraceae_Subgroup_3	Bryobacter	bOtu001439	61	Yes
	Actinobacteria	Actinobacteria	Frankiales	Acidothermaceae	Acidothermus	bOtu000037	59	Yes
	Planctomycetes	Planctomycetacia	Isosphaerales	Isosphaeraceae	unassigned	bOtu000704	59	Yes

SUPPLEMENTARY METHODS

Determination of soil physicochemical properties

In July 2023 (summer) and January 2024 (winter), we randomly selected five trees per plot as the sampling objects. Four soil cores were collected from the 0-30 cm root zone in the east, south, west, and north directions of the selected tree trunks using an undisturbed soil sampler (inner diameter: 5 cm), and the soil cores collected from the same plot were mixed together to form one sample. Overall, twenty-four samples (four treatments × three replicates × two seasons) were collected for the determination of physiochemical properties after air drying.

Soil water content (SWC) was determined by the drying method (105℃, 24h). Soil bulk density (SBD) was determined using the cutting ring method to calculate the dry weight per unit volume of soil. A soil: water (1:5 w/v) suspension was shaken violently for 2 min and allowed to stand for 30 min to determine pH by a pH meter (LEICI, China). Soil temperature (ST) was measured by a Thermochron iButton Device (DS1921-G, Maxim Integrated, San Jose, CA, United States). Soil organic carbon (SOC) was measured by wet oxidation with potassium[1]. The total nitrogen (TN) content was determined using the Kjeldahl method[2]. Total phosphorus (TP) was measured using the alkali fusion-Mo-Sb anti spectrophotometric method[3]. The total potassium (TK) content was determined using the Bao method[4].

SUPPLEMENTARY REFERENCES

[1] Lu, R. *Soil Agrochemical Analysis Methods*; China Agricultural Science and Technology Press: Beijing, China, 2000.
[2] Stanford, G.; Carter, J. N.; Simpson, E. C.; Schwaninger, D. E. Nitrate determination by a modified conway microdiffusion method. *J. AOAC Int.* **1973**, *56*, 1365-1368, doi: 10.1093/jaoac/56.6.1365.
[3] Ren, Z.; Qu, X.; Peng, W.; Yu, Y.; Zhang, M. Functional properties of bacterial communities in water and sediment of the eutrophic river-lake system of Poyang Lake, China. *PeerJ* **2019**, *7*, e7318. doi: 10.7717/peerj.7318.
[4] Bao, S. D. *Soil and Agricultural Chemistry Analysis*; China Agricultural Press: Beijing, China, 2000.
